# Macrophages with reduced expressions of classical M1 and M2 surface markers in human bronchoalveolar lavage fluid exhibit pro-inflammatory gene signatures

**DOI:** 10.1038/s41598-021-87720-y

**Published:** 2021-04-15

**Authors:** Hiroto Takiguchi, Chen X. Yang, Cheng Wei Tony Yang, Basak Sahin, Beth A. Whalen, Stephen Milne, Kentaro Akata, Kei Yamasaki, Julia Shun Wei Yang, Chung Yan Cheung, Ryan Vander Werff, Kelly M. McNagny, Fernando Sergio Leitao Filho, Tawimas Shaipanich, Stephan F. van Eeden, Ma’en Obeidat, Janice M. Leung, Don D. Sin

**Affiliations:** 1 St Paul’s Hospital, The University of British Columbia (UBC) Centre for Heart Lung Innovation (HLI), Vancouver, BC Canada; 2 The Biomedical Research Centre, University of British Columbia, Vancouver, BC Canada; 3 Division of Respiratory Medicine, UBC Department of Medicine, Vancouver, BC Canada; 4 Division of Pulmonary Medicine, Department of Medicine, Tokai University School of Medicine, Isehara, Kanagawa Japan; 5 Faculty of Medicine and Health, The University of Sydney, Sydney, NSW Australia

**Keywords:** Cell biology, Genetics, Immunology, Diseases

## Abstract

The classical M1/M2 polarity of macrophages may not be applicable to inflammatory lung diseases including chronic obstructive pulmonary disease (COPD) due to the complex microenvironment in lungs and the plasticity of macrophages. We examined macrophage sub-phenotypes in bronchoalveolar lavage (BAL) fluid in 25 participants with CD40 (a M1 marker) and CD163 (a M2 marker). Of these, we performed RNA-sequencing on each subtype in 10 patients using the Illumina NextSeq 500. Approximately 25% of the macrophages did not harbor classical M1 or M2 surface markers (double negative, DN), and these cells were significantly enriched in COPD patients compared with non-COPD patients (46.7% vs. 14.5%, *p* < 0.001). 1886 genes were differentially expressed in the DN subtype compared with  all other subtypes at a 10% false discovery rate. The 602 up-regulated genes included 15 mitochondrial genes and were enriched in 86 gene ontology (GO) biological processes including inflammatory responses. Modules associated with cellular functions including oxidative phosphorylation were significantly down-regulated in the DN subtype. Macrophages in the human BAL fluid, which were negative for both M1/M2 surface markers, harbored a gene signature that was pro-inflammatory and suggested dysfunction in cellular homeostasis. These macrophages may contribute to the pathogenesis and manifestations of inflammatory lung diseases such as COPD.

## Introduction

Macrophages are the most abundant immune cells in the lower airways of the human respiratory tract. They are involved in host immune defenses and airway homeostasis. They are also highly plastic, being able to change their phenotype and function depending on the local milieu^1,2^. Over the past few decades, a conceptual framework has evolved to describe the activation pattern of macrophages in vitro, which has been used to categorize macrophages into at least two distinct phenotypes: classically (M1) or alternatively (M2) activated macrophages^3^.

Briefly, classical activation of M1 macrophages is typically induced by lipopolysaccharide (LPS)/interferon (IFN)-γ or tumor necrosis factor (TNF) and contributes to a pro-inflammatory milieu, and demonstrate strong bactericidal activities, along with expression of CD40, CD80, CD86 and inducible nitric oxide synthase (iNOS). In contrast, alternatively activated M2 macrophages are stimulated by interleukin (IL)-4, and IL-13 and contribute to immunomodulation by scavenging debris, enabling tissue repair and promoting remodeling of local tissue. M2 macrophages are also characterized by a high expression level of scavenger receptors such as CD163 and CD206^4–6^. In general, M1 macrophages are thought to be pro-inflammatory, while the M2 macrophages limit inflammation and promote healing and homeostasis^7^. Although this simple classification works in vitro, it is of limited use in vivo. Owing to repeated exposures to external microbes and environmental toxins, the microenvironment in the lung can rapidly change, leading to modulations in the host immune response. Consistent with this notion, one study showed that chronic inflammation can lead to broad changes in the transcriptional repertoire of macrophages beyond the classical M1 and M2 phenotypes^8^. As there is a scarcity of data on the state of polarization of alveolar macrophages in vivo, the utility and appropriateness of the classical M1/M2 categorization of macrophages in the human airways are uncertain. We hypothesized that while in health most macrophages can be categorized based on M1/M2 polarization, in inflammatory lung conditions such as chronic obstructive pulmonary disease (COPD), most cells will be non-typeable. To address this hypothesis, here, we phenotyped macrophages in human bronchoalveolar lavage (BAL) fluid by using classical cell surface markers: CD40 for M1 macrophages and CD163 for M2 macrophages^5,9–11^. This study was exploratory in nature and its main purpose was to characterize the polarization and associated gene signatures of macrophages from human BAL fluid in health and disease according to their classical cell surface markers for M1/M2 phenotypes.

## Results

### Macrophage sub-phenotyping

We first performed flow cytometry on macrophages that were isolated in BAL fluid of 25 participants including 8 chronic obstrutive pulmonary disease (COPD) and 13 asthma patients and divided the cell population into four groups: double negative (DN), double positive (DP), and M1 and M2 based on cell surface markers. The mean percentage of DN, DP, M1 and M2 subtypes in the BAL fluid was 24.8%, 35.4%, 14.0% and 25.9%, respectively.

### RNA-sequencing

Next, we performed RNA-sequencing on BAL macrophages collected from 10 consecutive participants between March 2019 and June 2019. After exclusion of 3 samples (i.e. 1 DN and 2 DP’s, which were obtained from three patients with asthma) because of poor RNA quality or low RNA yield, we subjected the remaining samples (n = 37) to bulk-RNA sequencing. The demographic data of the study participants are summarized in Table S1.

#### Differentially expressed genes (DEGs) between subtypes

**Figure 1:**
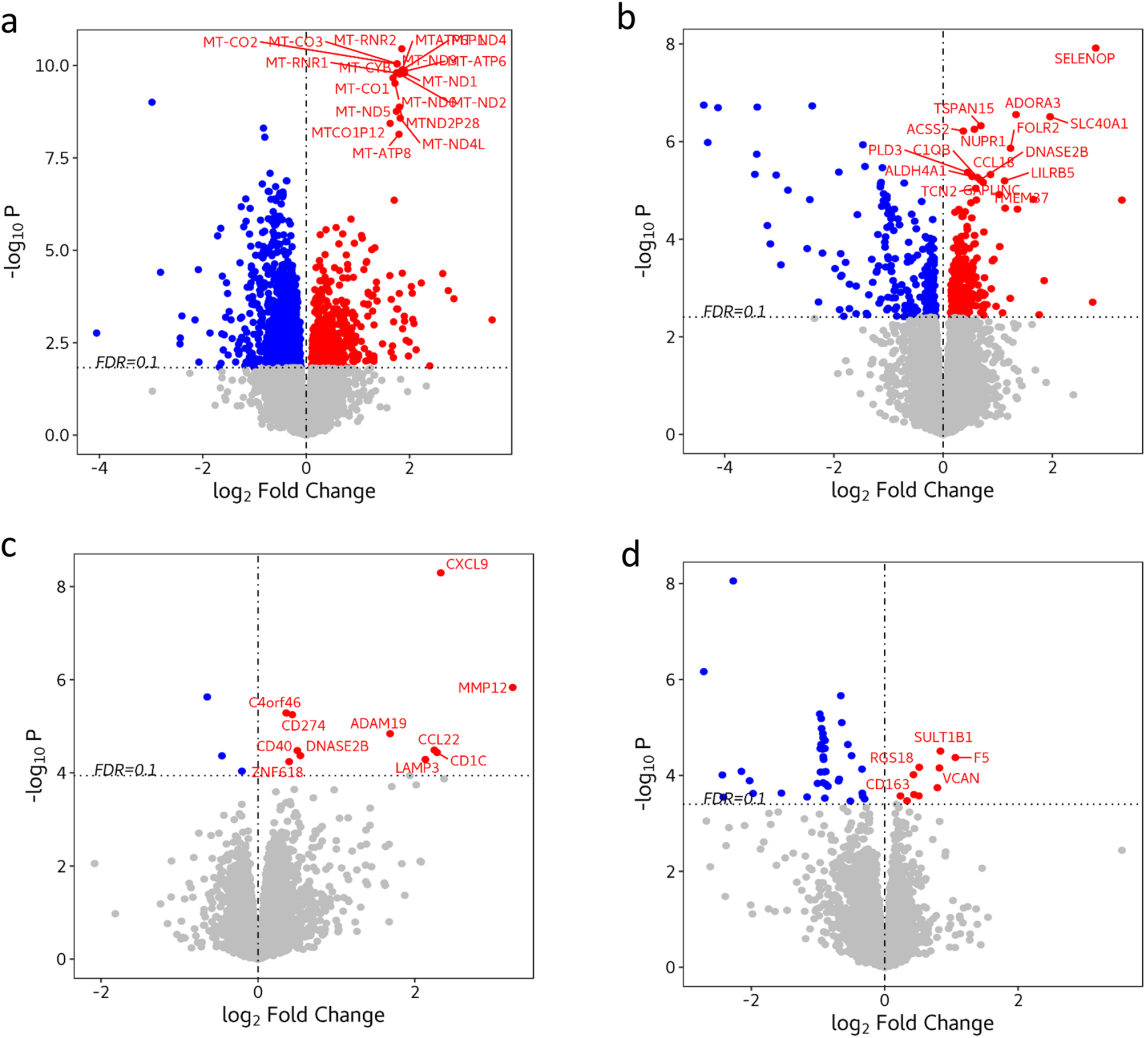
Volcano plots showing differentially expressed genes across macrophage subtypes (n = 10). (**a**) Double negative (DN) subtype versus the other subtypes. (**b**) Double positive (DP) subtype versus the others. (**c**) M1 subtype versus the others. (**d**) M2 versus the others. The plot shows the fold-change on the X-axis versus the unadjusted *p* values (on a –log_10_ scale) on the Y-axis. Differentially expressed genes at 10% FDR are represented as colored dots and the top 20 up-regulated genes for each cell-type are labelled on the graph. The greatest number of differentially expressed genes was observed with the DN subtype (1886 differentially expressed genes versus all other subtypes at 10% FDR) followed by 498 differentially expressed genes between the DP subtype and the others; 15 genes between the M1 subtype and the others; and 52 genes between the M2 subtype and the others. The top 20 up-regulated genes for the DN macrophages included 15 mitochondrial genes and 4 mitochondrial pseudogenes. Figure created with the R (version 3.5.0). https://www.r-project.org/.

We compared the transcriptomic expression pattern across all four subtypes of macrophages. The greatest number of differentially expressed genes was observed with the DN subtype (1886 differentially expressed genes versus all other subtypes at a 10% false discovery rate, FDR). There were 498 differentially expressed genes between the DP subtype and the others; 15 genes between the M1 subtype and the others; and 52 genes between the M2 subtype and the others (Fig. 1). All differentially expressed genes at 10% FDR are shown in Table S2.

Among the differentially expressed genes, 602 were up-regulated in the DN subtype, 292 were up-regulated in DP, 12 were up-regulated in M1 and 11 were up-regulated genes in the M2 subtype. The top 20 up-regulated genes for each subtype are shown in Fig. 1. Importantly, the top 20 up-regulated genes for the DN macrophages included 15 mitochondrial genes and 4 mitochondrial pseudogenes; for other macrophage subtypes, these mitochondrial genes were not differentially expressed.

**Figure 2:**
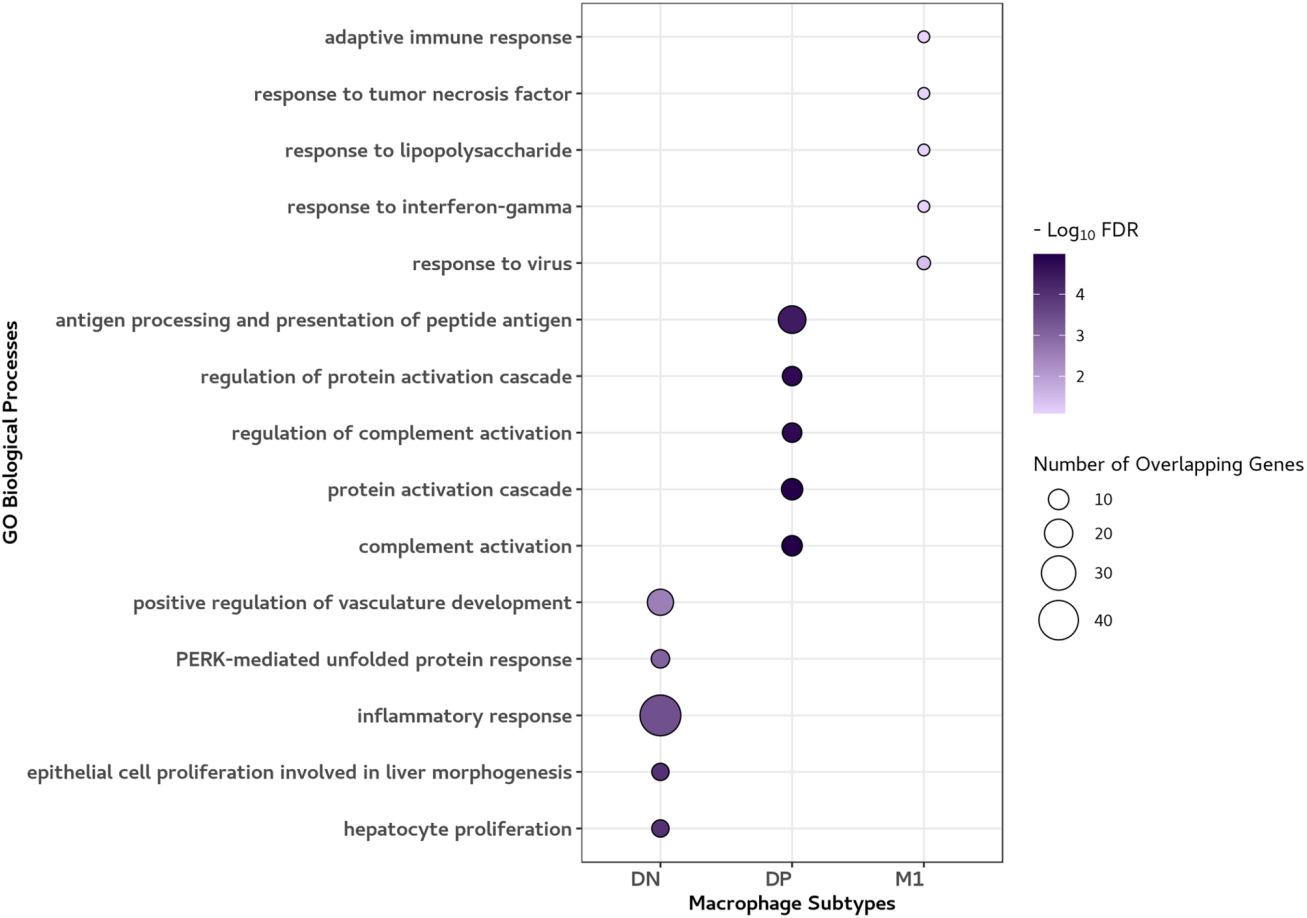
Enrichment analysis: The top 5 Gene Ontology (GO) biological processes based on up-regulated differentially expressed genes. The top 5 GO biological processes at 10% FDR for each macrophage subtype are shown. The circle size represents the number of overlapping genes between each GO process and up-regulated genes according to the subtype. The colour scale represents the extent to which the up-regulated genes are significantly enriched in each GO process. The GO processes associated with DN, DP and M1 subtype included inflammatory response, complement activation and response to virus, respectively, whereas none of the up-regulated genes for M2 macrophages were enriched in the GO processes. Figure created with the R package ggplot2. https://cran.r-project.org/web/packages/ggplot2/index.html.

Enrichment analysis was performed using only the up-regulated genes for each macrophage subtype at 10% FDR. Up-regulated genes in DN were enriched in 86 GO biological processes. The results of the enrichment analysis including the top 5 strongest *p*-values are shown in Fig. 2. These included genes involved in inflammatory responses (FDR = 3.89E−04).

For the DP subtype, up-regulated genes were enriched in 61 GO processes, which included pathways for complement activation (FDR = 1.05E−05), protein activation cascade (FDR = 1.05E−05), antigen processing and presentation of peptide antigen (FDR = 3.73E−05). Up-regulated genes in M1 were enriched in 21 GO processes such as responses to viruses (FDR = 3.79E−02), IFN-γ (FDR = 7.32E−02) and LPS (FDR = 7.70E−02). However, none of the up-regulated genes for M2 macrophages were enriched in the GO processes. All enriched GO biological processes at 10% FDR are shown in Table S3.

Likewise, we compared the transcriptomic expression pattern on the basis of CD163 positivity. Among 128 differentially expressed genes at 10% FDR, 39 genes were up-regulated and 89 genes were down-regulated in macrophages positive for CD163 (Fig. S1). All differentially expressed genes at 10% FDR are shown in Table S4. However, none of the up- or down-regulated genes were enriched in GO biological processes at 10% FDR.

#### Weighted gene co-expression network analysis (WGCNA)

Based on detectable expression levels of all genes on the RNAseq platform (18,314 genes in total), 13 gene expression modules were constructed using a weighted gene co-expression network analysis (WGCNA). The WCGNA-derived modules ranged in size from 19 genes in module 13 to 1,831 genes in module 1. The 13 modules as well as the “garbage module” (i.e. module 00) derived from WGCNA are shown in Table S5.

**Figure 3:**
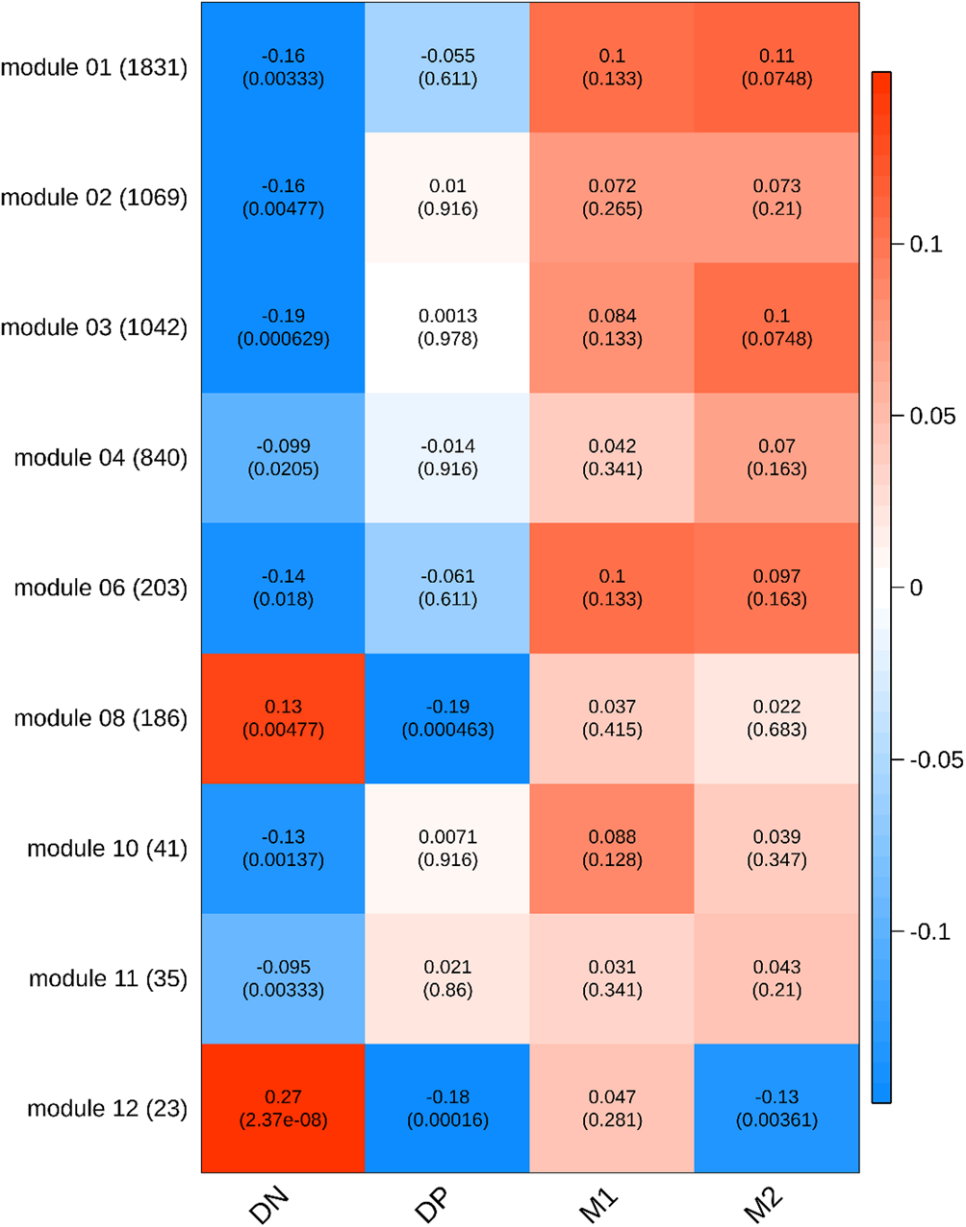
Heat map of the correlation of weighted gene co-expression network analysis (WCGNA) modules with macrophage subtypes (n = 10). The rows represent the gene modules and the sizes of the modules are shown in parentheses next to the module name. The columns represent macrophage subtypes. In each cell, the number at the top is the linear regression coefficient and the number in the parentheses is the corresponding *p*-value. Color scale represents the regression coefficient. Only modules with at least one significant cell at 10% FDR across four subtypes are  shown. Gene signatures in the DN subtype were significantly distinct from those of the othersubtypes . Importantly, Module 12, which was composed of 15 mitochondrial genes, was up-regulated in DNs, and was down-regulated in the DP and M2 subtypes. Figure created with the R package “WGCNA” (version 1.68). https://cran.r-project.org/web/packages/WGCNA/index.html.

Among these module eigengenes, nine of them were differentially expressed in at least one macrophage subtype at a 10% FDR threshold (Fig. 3). Gene signatures in the DN subtype were significantly distinct from those of other subtypes. In contrast, M1 macrophages were not associated with a distinct module. Importantly, module 12, which was up-regulated in DNs, was down-regulated in the DP and M2 subtypes. This module was composed of 15 mitochondrial genes, 13 protein subunit genes, 2 ribosomal RNA and 4 mitochondrial pseudogenes. The top genes with the highest membership in this module were *MT-ATP6*, *MT-CYB* and *MT-ND4*.

**Figure 4:**
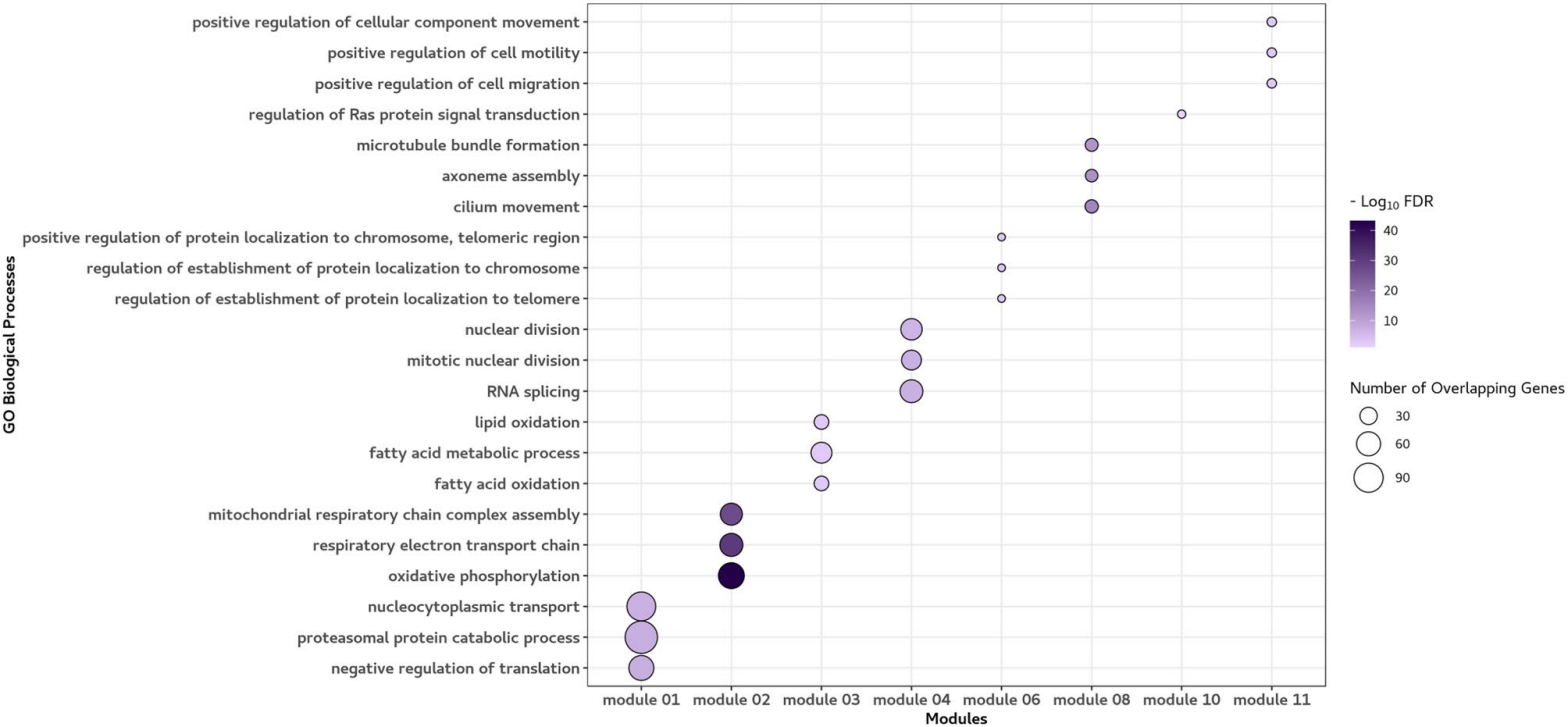
The top 3 GO biological processes at 10% FDR based on a weighted gene co-expression network analysis (WGCNA). The top 3 GO biological processes at 10% FDR for each module are shown. The circle size represents the number of overlapping genes between each GO process and genes in the module. The colour scale represents the extent to which the genes are significantly enriched in each module. Module 2 was most strongly associated with the function of ATP production in mitochondria. Module 3 was  associated with fatty oxidation and metabolism. Module 4 was associated with RNA splicing and mitotic nuclear division. Module 6 was associated with regulation of protein localization to telomeres. Figure created with the R package ggplot2. https://cran.r-project.org/web/packages/ggplot2/index.html.

Enrichment analysis was performed at 10% FDR to identify GO biological processes that were associated with these modules. The top three GO processes are shown in Fig. 4. Module 2 was associated with the function of ATP production in mitochondria including oxidative phosphorylation (FDR = 6.32E−44) and respiratory electron transport chain (FDR = 2.62E−31). Module 3 was associated with fatty oxidation and metabolism (FDR = 1.49E−03). Module 4 was associated with RNA splicing (FDR = 4.88E−08) and mitotic nuclear division (FDR = 4.88E−08). Module 6 was associated with regulation of protein localization to telomeres (FDR = 2.81E−04). Mitochondrial genes in module 12 were not included in the enrichment analysis because of the absence of a reference gene set for these genes. All enriched GO biological processes at 10% FDR and the top 20 genes with the highest membership for each of the modules are shown in Tables S6 and S7, respectively.

**Figure 5:**
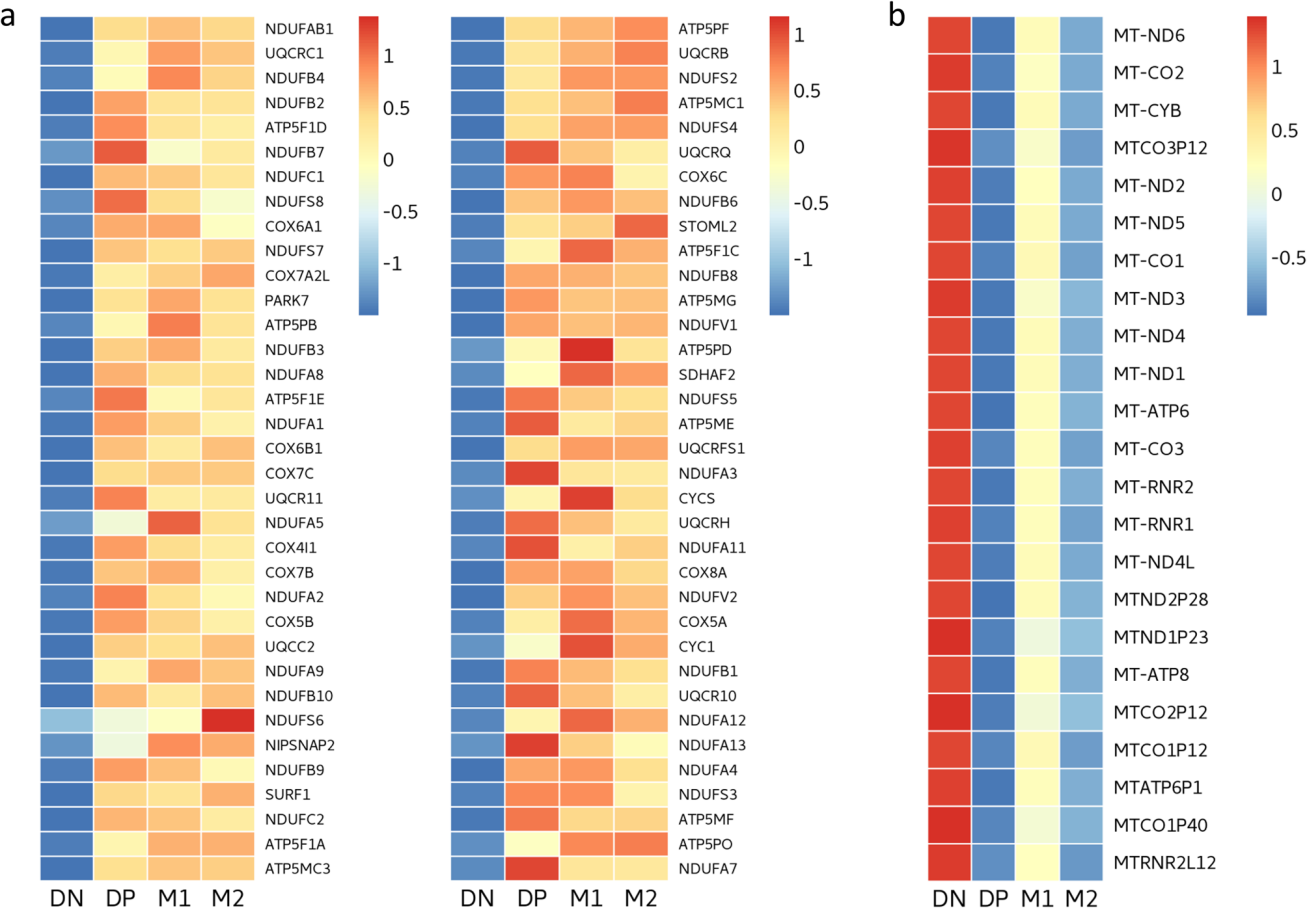
Relative gene expression across macrophage subtypes (n = 10). Heat maps showing (**a**) genes in the gene ontology (GO) biological process of oxidative phosphorylation overlapping with genes in module 2 and (**b**) mitochondrial genes in module 12. Color scale represents the scaled mean expression level (log_2_ TPM) of each subtype (red indicates up-regulation, blue indicates down-regulation). The oxidative phosphorylation genes were down-regulated in the DN subtype, whereas the mitochondrial genes were up-regulated in the DN subtype. Figure created with the R package “NMF” (version 0.22.0). https://cran.r-project.org/web/packages/NMF/index.html.

To further explore potential changes in mitochondrial respiration in the DN subtype, we examined the relative gene expression in the GO biological process of oxidative phosphorylation, which overlapped with those in module 2, and the mitochondrial genes in module 12. The oxidative phosphorylation genes were down-regulated in the DN subtype (Fig. 5a), whereas the mitochondrial genes were up-regulated in the DN subtype (Fig. 5b).

### Macrophage sub-phenotype distributions across diseases

Among 25 participants including 8 with COPD and 13 with asthma, we investigated the distribution of macrophage subtypes based on disease. Because the presence of asthma did not significantly alter the macrophage subtype distribution (Fig. S2), we compared the subtypes between participants with and without COPD. Patients with COPD were more likely to be males (*p* = 0.003) and current smokers (*p* = 0.001) and had lower FEV_1_/FVC (*p* = 0.006). None of the patients with COPD had been previously diagnosed with asthma. A majority of patients with COPD had moderate to severe airflow limitation. Baseline characteristics are shown in Table 1.

**Table 1:** The demographic data of patients with or without chronic obstructive lung disease (COPD).

	COPD	non-COPD	*p* value
	n = 8	n = 17	
Age, years	59.4 ± 11.3	61.2 ± 8.6	0.65
Male	8 (100)	6 (35.3)	0.003
**Ethnicity**			0.74
Caucasian	6 (75.0)	14 (82.4)	
Others^a^	2 (25.0)	3 (17.6)	
Body mass index, kg/m^2^ (n = 23)	24.0 ± 4.5	27.7 ± 9.1	0.3
**Smoking status (n = 24)**			0.001
Current	4 (57.1)	0 (0.0)	
Former	3 (42.9)	7 (41.2)	
Never	0 (0.0)	10 (58.8)	
Pack-year smoked (n = 13)	32.6 ± 23.1	43.5 ± 56.3	0.65
**Pulmonary function test (n = 24)**			
FEV_1_/FVC, %	55.3 ± 15.5	69.5 ± 7.6	0.006
FEV_1_, % predicted	59.4 ± 23.6	73.9 ± 22.9	0.16
FVC, % predicted	87.5 ± 18.4	82.1 ± 20.6	0.54
**GOLD stage**			
Stage 1/2	1 (12.5)/5 (62.5)	NA	
Stage 3/4	1 (12.5)/1 (12.5)	NA	
Asthma	0 (0.0)	13 (76.5)	< 0.001
**Pharmacotherapy**			
Inhaled corticosteroid	1 (12.5)	7 (41.2)	0.21
Oral corticosteroid	0 (0.0)	3 (17.6)	0.53
**Macrophage subtype**			
Double negative	46.7 ± 27.6	14.5 ± 9.2	< 0.001
Double positive	16.5 ± 12.0	44.3 ± 19.5	0.001
M1	14.0 ± 14.1	14.0 ± 18.5	0.99
M2	22.8 ± 22.2	27.3 ± 19.8	0.61

Values are means ± standard deviation (SD) or numbers (%) of observations. Continuous and categorical variables are compared using a Student’s t-test or a Fisher’s exact test, respectively.

*COPD* Chronic obstructive pulmonary disease, *FEV*_*1*_ Forced expiratory volume in 1 s, *FVC* Forced vital capacity, *GOLD* The Global Initiative for Chronic Obstructive Lung Disease, *NA* Not applicable. GOLD grade was defined as grade 1: FEV_1_ ≥ 80% predicted; grade 2: FEV_1_ 50–79% predicted; grade 3: FEV_1_ 30–49% predicted; grade 4: FEV_1_ < 30% predicted.

^a^Other ethnicity included Asians, African Canadians, and First nations.

Macrophages in patients with COPD were less likely to express classical surface markers including CD 163 compared to those without COPD (39.3% vs. 71.6%, *p* = 0.004). For the four macrophage sub-phenotypes, the DN subtype was enriched in patients with COPD (46.7% vs. 14.5%, *p* < 0.001). In contrast, the DP subtype was significantly reduced in COPD (16.5% vs. 44.3%, *p* = 0.001). There were no significant differences in terms of M1 and M2 subtypes (Table 1).

## Discussion

Here, we showed that many macrophages in human BAL did not conform to the M1/M2 paradigm, and that these cells, especially those that did not harbor M1 or M2 cell surface markers, demonstrated distinct transcriptomic signatures. Interestingly, the double negative subtype contained differential expression of mitochondrial genes, which were significantly enriched in patients with COPD. Although previous studies have attempted to characterize the gene signatures in the framework of dichotomized classification in vitro, or in vivo, to the best of our knowledge, this is the first study to shed light on the transcriptional profile of macrophages beyond the M1/M2 classification in human BAL fluid^12–14^.

To date, the prevalence of M1 and M2 macrophages in vivo has not been well characterized. A priori we decided to use CD40 and CD163 based on previous studies, which showed that they were distinct markers for human M1 and M2 macrophages^9–11^. A number of studies have shown that CD40 and CD163 are expressed on 4–20% and 45–60%, respectively, of human lung macrophages in non-smoking individuals^9,15,16^. We extend these findings by showing that approximately 25% of all macrophages in human BAL fluid could not be phenotyped with CD40 and CD163 (and thus were deemed “double negative” macrophages); and that the percentage of these double negative cells increased significantly in BAL fluid of patients with COPD. This observation is consistent with previous studies which reported reduced expression of CD163 and CD40 on alveolar macrophages in COPD lungs^9,17^.

We also showed that these double negative cells demonstrate a distinct RNA signature compared with M1, M2 or double positive macrophages. The up-regulated, differentially expressed genes in double negatives were significantly enriched in inflammatory responses. The GO inflammatory responses were relatively broad and contained 43 genes including *NFKB1* (NFκB1), *NFKB2* (NFκB2), *NFKBIA* (IκBα), *TNFRSF1B* (TNFR2), *CXCL1*, *CXCL2*, *CXCL8*, *IL1B* (IL-1β) and *NLRP3*. The transcription factors NF-κB and TNF orchestrate many immune and inflammatory responses including stress responses and regulation of cell proliferation and apoptosis^18–20^. CXCL1, CXCL2, CXCL8 and IL-1β are powerful pro-inflammatory cytokines released by macrophages for neutrophil recruitment^21–23^. NLRP3, on the other hand, has been linked with age-related cellular dysfunction, or “inflammaging”, which is characterized by dysregulated low-grade inflammation^24^.

In WGCNA, interestingly, we found that module 2, which was associated with oxidative phosphorylation in mitochondria, was down-regulated in double negative macrophages. Mitochondria is the main producer of cellular energy by means of oxidative phosphorylation, which involves electron-transferring respiratory chain (complexes I–IV) and adenosine triphosphate (ATP) synthase (complex V). Although mitochondria has its own DNA, which contains 37 genes, over 98% of the mitochondrial proteins are encoded by the nuclear genome^25,26^. We found that a number of nuclear genes, which control cellular respiration and ATP synthesis in the mitochondria, were markedly down-regulated in double negative macrophages. For instance, 32 genes encoding the NADH: ubiquinone oxidoreductase supernumerary subunits (NDUF), which form an essential component of complex I of the respiratory chain, were depleted in double negative macrophages compared to the other subtypes, suggesting a reduced ability to generate ATP on demand to maintain cellular functions^27^. On the other hand, we observed a significant up-regulation of 15 mitochondrial genes in double negative macrophages in module 12.

The origins of double negative macrophages are unknown. Previous studies have shown that aged cells have reduced expression of surface markers including MHC class II molecules and co-stimulatory receptors such as CD40, which raises the possibility that the double negative macrophages may be senescent cells^28,29^. This notion is further supported by our data showing that these cells harbor differentially expressed genes involved in inflammation and mitochondrial (dys)function^30^. In the present study, we could not distinguish tissue resident macrophages from monocyte-derived macrophages^31^. It is noteworthy, however, *MARCO* was down-regulated in double negative macrophages (logFC = − 0.35, FDR = 0.021) compared to the other subtypes. MARCO is a marker of embryonically-derived resident macrophages, which raises the possibility that many of the double negatives originated from monocyte-derived macrophages^32^. Specific studies focused on cell lineages will be needed to pinpoint the exact source of these and other macrophage subtypes.

There were several limitations in this study. First, some of the up-regulated genes in M1 or M2 macrophages in our study did not fully align with classical patterns associated with M1- or M2-related genes. For instance, up-regulated genes in M1 macrophages included those encoding for proteins such as CCL22 and MMP12, which have been related to M2 macrophages^33,34^. Also, genes encoding CD40 and CD163 did not co-express with genes encoding other surface markers commonly used to characterize M1 or M2 macrophages such as CD80 or CD206, respectively^5^. However, these markers were identified using in vitro generated macrophages where M1-related markers were induced by LPS/IFN-γ and M2-related markers were induced by IL-4/IL-13. A comparative study of in vivo and in vitro macrophages showed that the gene signature of M1 macrophages activated by LPS in vivo shared many features of alternatively activated M2 genes in vitro including up-regulation of CCL22^35^. While not all M1 and M2 markers in vitro can be directly translated to the in vivo situation^35^, further studies are needed to validate our findings with other M1 and M2 markers  using technologies such as flow cytometry and single cell sequencing. Second, although the unique gene expression signature of double negative macrophages was interesting, we did not conduct functional studies to validate the potential mitochondrial dysfunction in these macrophages, which were suggested by the RNA sequencing data. Third, it is possible that some of our samples may have contained dendritic cells as they have similar morphology and share common surface markers such as HLA-DR and CD40 as macrophages^36^. Although dendritic cells generally constitute only 0.5% of total cells in BAL fluid, future studies should gate out these cells for more precise assessment of alveolar macrophages^37,38^.

In conclusion, approximately one out of four macrophages in human BAL fluid stain negatively for both M1/M2 cell surface markers. These double negative macrophages harbor gene expression signature that is pro-inflammatory and suggests dysfunction in cellular metabolism and homeostasis. These cells increase by threefold in the BAL fluid of COPD patients. Together, these data suggest that phenotypic shifts in alveolar macrophages may play a significant role in the pathogenesis and disease manifestations of inflammatory disease conditions such as COPD.

## Methods

### Study population

Following informed consent, we performed bronchoscopy and collected bronchoalveolar lavage (BAL) fluid in 25 patients including those with COPD, who participated in a clinical trial: Differential Effects of Inhaled Symbicort and Advair on Lung Microbiota (DISARM) (ClinicalTrials.gov identifier: NCT02833480) or those who underwent clinical bronchoscopy because of a pulmonary nodule between November 2017 and June 2019 at St. Paul’s Hospital (SPH) in Vancouver, Canada. These studies were approved by the University of British Columbia Clinical Research Ethics Board (certificate numbers: H14-02277 and H15-02166) and were conducted in accordance with the principles of the Declaration of Helsinki. COPD was defined based on the Global Initiative for Chronic Obstructive Lung Disease (GOLD) recommendations: symptoms of cough, dyspnea or sputum production, ≥ 10 pack-years of smoking history and a post-bronchodilator FEV_1_/FVC < 70% of predicted^39^. Asthma was defined based on the Global Initiative for Asthma (GINA) guidelines: symptoms of wheeze, cough or shortness of breath and airflow limitation that varied over time in individuals with a smoking history of less than 5 pack-years^40^. Patients were clinically stable and those who experienced an exacerbation of their COPD or asthma within at least four weeks of bronchoscopy were excluded.

### Bronchoscopy procedure

All procedures were performed by an experienced pulmonologist. Conscious sedation was first provided to the patient with the use of intravenous midazolam and fentanyl. Through the working channel of the bronchoscope (Olympus Corporation, Tokyo, Japan), topical 2% lidocaine was instilled, if needed, to prevent bronchospasm and cough. BAL was generally taken from the right middle lobe or lingula unless these lobes had disease noted on a pre-operative computed tomography (CT) imaging (e.g. pulmonary nodule). For these cases, the right or left upper lobe was used. After the bronchoscope was fully wedged into the desired segment, 20 ml of 0.9% saline was instilled (with a dwell time of 10 s) and then the fluid was manually aspirated out using a vacuum syringe. The first aliquot of the recovered solution was discarded because of concerns over contamination by bronchial lining fluid or mucus secretions. Aliquots of 40 ml of saline were then sequentially instilled to a maximum volume of 200 mL or until 30-50 mL of BAL fluid was recovered, whichever came first.

### Sample preparation

The recovered BAL fluid was put in ice immediately after aspiration and filtered through sterile 70 µm DNase/RNase-free cell strainers to remove large clumps and debris. Cells were recovered by centrifugation at 500 g for 10 min at 4 °C and were washed twice with PBS. The concentration of viable cells was determined by trypan blue staining on a hemocytometer. These samples generally contained 5 × 10^5^ to 5 × 10^6^ viable cells in 100uL of solution. These samples were then blocked with 10% human serum for 20 min and then stained with the following monoclonal antibodies against surface receptors (Biolegend, San Diego, US): anti-human HLA-DR antibody APC/Cy7 (Cat# 307,618, RRID: AB_493586), anti-human CD40 antibody Brilliant Violet 421 (Cat# 334,332, RRID: AB_2564211) and anti-human CD 163 Alexa Fluor 647 (Cat# 333,620, RRID: AB_2563475). The isotype control and compensation control for anti-human HLA-DR antibody APC/Cy7 were prepared in 2 separate tubes. Cells were incubated with the antibodies for 30 min, re-suspended and then incubated for an additional 30 min. At the end of the incubation period, the samples were washed in PBS and re-suspended in 600uL of PBS. Prepared samples were then analyzed using flow cytometry.

### Flow cytometry and cell sorting

Flow cytometry was performed on a MoFlo Astrios-EQ cell sorter (Beckman Coulter, Brea, US). Single macrophage was gated by the forward and side scatter and the presence (or absence) of HLA-DR. Subsequently, the macrophage population was examined with CD40 (a M1 marker) or CD163 (a M2 marker). We decided to use respective isotype controls for dividing the CD40/CD163 plot because their colors were separated by both the laser line and the emission spectra, while we used an anti-human HLA-DR antibody APC-Cy7 single stain to adjust for spillover into anti-human CD163 Alexa Fluor 647. Positive expression of each marker was determined at > 2% compared to its isotype control. This procedure resulted in cells being grouped into 4 categories: double negative (DN)—CD40−/CD163−; double positive (DP)—CD40+/CD163+; M1—CD40+/CD163−; and M2—CD40−/CD163+. All flow cytometry data were analyzed using the Kaluza analysis software (Beckman Coulter, Brea, US). Representative flow cytometry panels are shown in Fig. S3. Sorted cells were collected directly into a lysis buffer (350uL of Buffer RLT Plus). The Buffer RLT Plus was supplemented with 1% 2-mercaptoethanol as suggested by the manufacturer of the Allprep DNA/RNA Mini kit (Qiagen, Hilden, Germany). All samples were thoroughly homogenized by vortexing for 90 s in the presence of the lysis buffer and then stored in -80C freezer and thawed once for RNA extraction.

#### RNA preparation for Illumina Next Seq

Total RNA was extracted using the Allprep DNA/RNA Mini kit. Sample quality control and sequencing was performed at the Biomedical Research Centre in University of British Columbia. Samples were evaluated for quality using Agilent 2100 Bioanalyzer (Agilent Technologies, Santa Clara, US) and those that passed the quality test were then prepped for sequencing using a standard protocol for the NEBnext Ultra ii Stranded mRNA (New England Biolabs, Ipswich, US). Sequencing was performed on Illumina NextSeq 500 with Paired End 42 bp × 42 bp reads.

### Statistical analysis and RNA sequencing data processing

All statistical analyses were performed in R. For patient characteristics, continuous data including the distribution of macrophage subtypes were represented as mean ± standard deviation (SD) and categorical data as numbers (%) of observations. Statistical significance between COPD and non-COPD patients was assessed using a student’s t-test for continuous variables, and a Fisher’s exact test for categorical variables. *P* values < 0.05 were considered significant.

In RNA-seq data processing, raw sequencing reads were quality controlled using FastQC^41^. STAR (Spliced Transcripts Alignment to a Reference) was used to align the reads to GENCODE GRCh37 (version 31) genome reference and RSEM (RNA-Seq by Expectation Maximization) was used for quantification to obtain the counts and the transcript per million (TPM)^42,43^. The principal component analysis was used to check for potential batch effect and confounding factors. No obvious batch effect was observed but smoking status showed a potential confounding effect on the gene expression data (Fig. S4). Limma voom was used to normalize the count to log_2_ counts per million (CPM)^44^. Log_2_ CPM was used for the differential expression analysis while TPM, which normalizes for gene-length was used for the weighted gene co-expression network analysis (WGCNA). Genes with low abundance (log_2_ CPM < 1 or TPM < 5 in more than one-fourth of the samples) were filtered out.

For differential expression analysis, limma’s mixed effect model with adjustment of smoking status was used to compare one macrophage subtype versus the rest of the subtypes to identify characteristic gene expression signatures for each macrophage subtype. The Benjamini–Hochberg procedure was used to correct for multiple hypothesis testing and to control the false discovery rate (FDR); 10% FDR was used in line with previous studies which also have used 10% FDR^45–47^. A Gene Ontology (GO) enrichment analysis was performed on the significantly up-regulated genes at 10% FDR for each macrophage subtype using the R package, clusterProfiler^48,49^.

For the weighted gene co-expression network analysis (WGCNA), the R package WGCNA was used to construct gene modules with genes that were co-expressed with each other. The R code for WGCNA is available at https://github.com/yyolanda/macrophage_rnaseq. Given that gene length may have an effect on gene clustering, the gene-length normalized TPM was used for WGCNA^48^. We chose a soft-thresholding power (β) of 29 to obtain a scale free topology model fit index (R^2^) of > 0.8 (Fig. S5). The minimum module size was set to 15 genes and modules with a distance < 0.2 were merged together. A signed network with thirteen modules, excluding the “garbage” module which contained genes that did not co-express with any other genes, was constructed and each module was assigned a number. The “garbage” module was discarded and not included in the downstream analysis. The eigengene of each module was obtained by calculating the first principal component of the module and was considered as a representative of the expression profile of the module. Figure S6 shows a dendrogram of the clustering of the module eigengenes and a heatmap of the correlation between the module eigengenes. Limma’s mixed effect model was used to identify the module eigengenes that were associated with each macrophage subtype after adjusting for smoking status. For modules that were significantly associated with any of the macrophage subtypes at 10% FDR, a Gene Ontology (GO) enrichment analysis was performed using the R package clusterProfiler to identify biological processes that were associated with the genes of each module. To further investigate these modules, we ranked the module genes by their module membership. The top genes with larger and positive module membership were highly connected to the other genes in the module and exhibited the same direction of effect as the module eigengene. As a sensitivity analysis, we performed the analyses using a 5% FDR; these results are shown in Tables S8, S9, S10 and S11.

## Supplementary Information

Supplementary Information 1.

Supplementary Information 2.

## Data Availability

The datasets used for the current study are available from the senior author (DDS) upon a reasonable request.

## References

[1] Gordon S, Martinez FO (2010). Alternative activation of macrophages: mechanism and functions. Immunity.

[2] Tarique AA (2015). Phenotypic, functional, and plasticity features of classical and alternatively activated human macrophages. Am. J. Respir. Cell Mol. Biol..

[3] Mills CD, Kincaid K, Alt JM, Heilman MJ, Hill AM (2000). M-1/M-2 macrophages and the Th1/Th2 paradigm. J. Immunol..

[4] Arora S, Dev K, Agarwal B, Das P, Syed MA (2018). Macrophages: their role, activation and polarization in pulmonary diseases. Immunobiology.

[5] Mantovani A (2004). The chemokine system in diverse forms of macrophage activation and polarization. Trends Immunol..

[6] Yamasaki K, Eeden SFV (2018). Lung macrophage phenotypes and functional responses: role in the pathogenesis of COPD. Int. J. Mol. Sci..

[7] Hussell T, Bell TJ (2014). Alveolar macrophages: plasticity in a tissue-specific context. Nat. Rev. Immunol..

[8] Xue J (2014). Transcriptome-based network analysis reveals a spectrum model of human macrophage activation. Immunity.

[9] Chana KK, Fenwick PS, Nicholson AG, Barnes PJ, Donnelly LE (2014). Identification of a distinct glucocorticosteroid-insensitive pulmonary macrophage phenotype in patients with chronic obstructive pulmonary disease. J. Allergy Clin. Immunol..

[10] Vogel DY (2014). Human macrophage polarization in vitro: maturation and activation methods compared. Immunobiology.

[11] Wojtan P, Mierzejewski M, Osińska I, Domagała-Kulawik J (2016). Macrophage polarization in interstitial lung diseases. Cent. Eur. J. Immunol..

[12] Beyer M (2012). High-resolution transcriptome of human macrophages. PLoS ONE.

[13] Gerrick KY (2018). Transcriptional profiling identifies novel regulators of macrophage polarization. PLoS ONE.

[14] Martinez FO, Gordon S, Locati M, Mantovani A (2006). Transcriptional profiling of the human monocyte-to-macrophage differentiation and polarization: new molecules and patterns of gene expression. J. Immunol..

[15] Kaku Y (2014). Overexpression of CD163, CD204 and CD206 on alveolar macrophages in the lungs of patients with severe chronic obstructive pulmonary disease. PLoS ONE.

[16] Nicod LP, Joudrier S, Isler P, Spiliopoulos A, Pache JC (2005). Upregulation of CD40, CD80, CD83 or CD86 on alveolar macrophages after lung transplantation. J. Heart Lung Transpl..

[17] Dewhurst JA (2017). Characterisation of lung macrophage subpopulations in COPD patients and controls. Sci. Rep..

[18] Aggarwal BB (2003). Signalling pathways of the TNF superfamily: a double-edged sword. Nat. Rev. Immunol..

[19] Baker RG, Hayden MS, Ghosh S (2011). NF-κB, inflammation, and metabolic disease. Cell Metab..

[20] Wajant H, Siegmund D (2019). TNFR1 and TNFR2 in the control of the life and death balance of macrophages. Front. Cell Dev. Biol..

[21] Carmi Y (2009). The role of macrophage-derived IL-1 in induction and maintenance of angiogenesis. J. Immunol..

[22] De Filippo K (2013). Mast cell and macrophage chemokines CXCL1/CXCL2 control the early stage of neutrophil recruitment during tissue inflammation. Blood.

[23] Kunkel SL, Standiford T, Kasahara K, Strieter RM (1991). Interleukin-8 (IL-8): the major neutrophil chemotactic factor in the lung. Exp. Lung Res..

[24] Lee J (2017). Study of the NLRP3 inflammasome component genes and downstream cytokines in patients with type 2 diabetes mellitus with carotid atherosclerosis. Lipids Health Dis..

[25] Ryan MT, Hoogenraad NJ (2007). Mitochondrial-nuclear communications. Annu. Rev. Biochem..

[26] Taylor RW, Turnbull DM (2005). Mitochondrial DNA mutations in human disease. Nat. Rev. Genet..

[27] Vinothkumar KR, Zhu J, Hirst J (2014). Architecture of mammalian respiratory complex I. Nature.

[28] Herrero C (2002). Immunosenescence of macrophages: reduced MHC class II gene expression. Exp. Gerontol..

[29] Li G, Smithey MJ, Rudd BD, Nikolich-Žugich J (2012). Age-associated alterations in CD8α+ dendritic cells impair CD8 T-cell expansion in response to an intracellular bacterium. Aging Cell.

[30] Youm YH (2013). Canonical Nlrp3 inflammasome links systemic low-grade inflammation to functional decline in aging. Cell Metab..

[31] Gundra UM (2014). Alternatively activated macrophages derived from monocytes and tissue macrophages are phenotypically and functionally distinct. Blood.

[32] Gibbings SL (2015). Transcriptome analysis highlights the conserved difference between embryonic and postnatal-derived alveolar macrophages. Blood.

[33] Nakagomi D (2015). Matrix metalloproteinase 12 is produced by M2 macrophages and plays important roles in the development of contact hypersensitivity. J Allergy Clin Immunol.

[34] Shaykhiev R (2009). Smoking-dependent reprogramming of alveolar macrophage polarization: implication for pathogenesis of chronic obstructive pulmonary disease. J. Immunol..

[35] Orecchioni M, Ghosheh Y, Pramod AB, Ley K (2019). Macrophage polarization: different gene signatures in M1(LPS+) vs. classically and M2(LPS-) vs. alternatively activated macrophages. Front. Immunol..

[36] Condon TV, Sawyer RT, Fenton MJ, Riches DW (2011). Lung dendritic cells at the innate-adaptive immune interface. J. Leukoc. Biol..

[37] Bratke K (2007). Dendritic cell subsets in human bronchoalveolar lavage fluid after segmental allergen challenge. Thorax.

[38] Tighe RM (2019). Improving the quality and reproducibility of flow cytometry in the lung. An Official American Thoracic Society Workshop Report. Am. J. Respir. Cell Mol. Biol..

[39] Vodermaier HC (2001). Cell cycle: waiters serving the destruction machinery. Curr. Biol..

[40] Bateman ED (2008). Global strategy for asthma management and prevention: GINA executive summary. Eur. Respir. J..

[41] Wingett SW, Andrews S (2018). FastQ screen: a tool for multi-genome mapping and quality control. F1000Res.

[42] Dobin A (2013). STAR: ultrafast universal RNA-seq aligner. Bioinformatics.

[43] Li B, Dewey CN (2011). RSEM: accurate transcript quantification from RNA-Seq data with or without a reference genome. BMC Bioinform..

[44] Law CW, Chen Y, Shi W, Smyth GK (2014). voom: precision weights unlock linear model analysis tools for RNA-seq read counts. Genome Biol..

[45] Anders S, Huber W (2010). Differential expression analysis for sequence count data. Genome Biol..

[46] Jaffe AE (2018). Developmental and genetic regulation of the human cortex transcriptome illuminate schizophrenia pathogenesis. Nat. Neurosci..

[47] Love MI, Huber W, Anders S (2014). Moderated estimation of fold change and dispersion for RNA-seq data with DESeq2. Genome Biol..

[48] Mandelboum S, Manber Z, Elroy-Stein O, Elkon R (2019). Recurrent functional misinterpretation of RNA-seq data caused by sample-specific gene length bias. PLoS Biol..

[49] Yu G, Wang LG, Han Y, He QY (2012). clusterProfiler: an R package for comparing biological themes among gene clusters. OMICS.

