# Supplementary material for: Macrophages with reduced expressions of classical M1 and M2 surface markers in human bronchoalveolar lavage fluid exhibit pro-inflammatory gene signatures
Source: Sci Rep. 2021 Apr 15;11:8282. doi: 10.1038/s41598-021-87720-y (PMC8050093; doi:10.1038/s41598-021-87720-y)
Supplement: Supplementary file 1 — Supplementary Information 1. [file 41598_2021_87720_MOESM1_ESM.pdf]

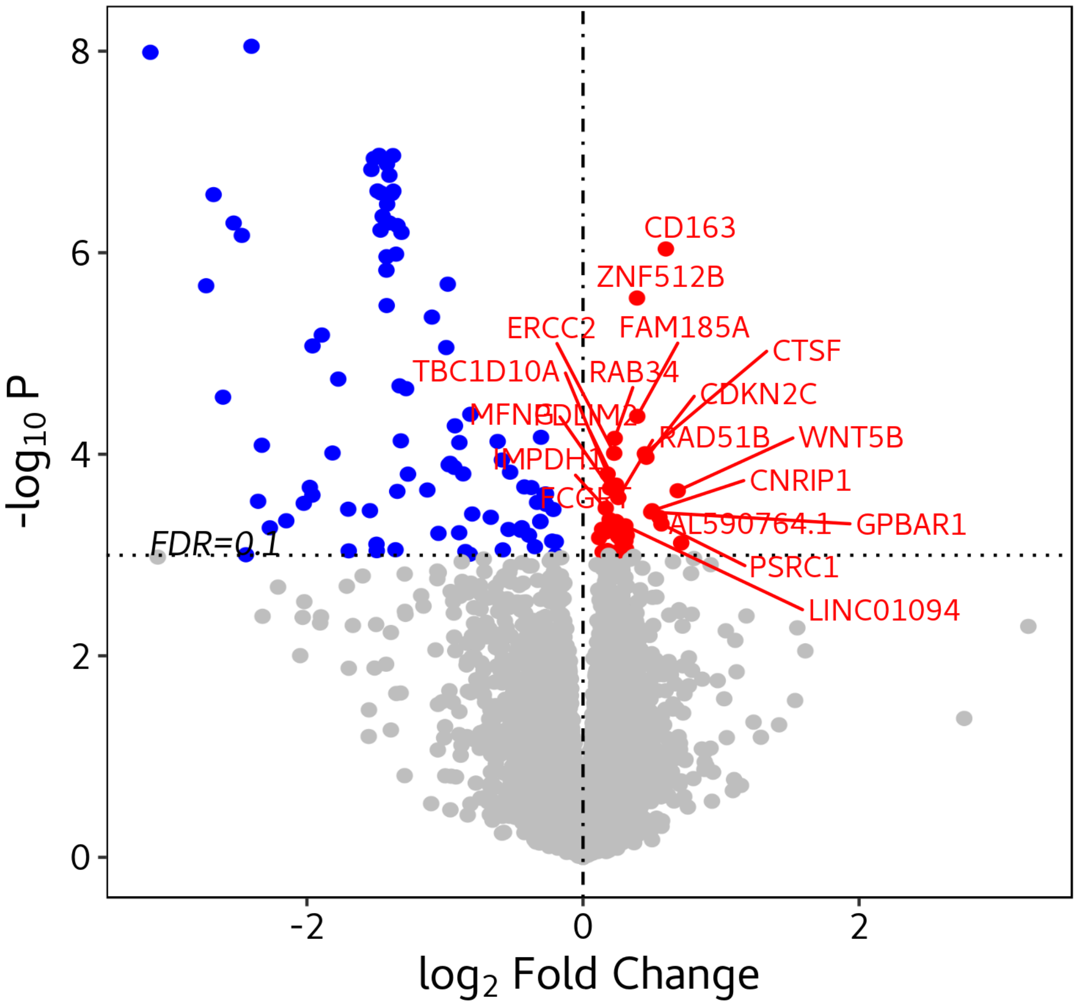

Figure S1. Volcano plots showing differentially expressed genes on the basis of CD 163 positivity (n = 10). The plot shows the fold-change on the X-axis versus the unadjusted p-values (on the–log10 scale) on the Y-axis. Differentially expressed genes at 10% FDR are represented as colored dots and the top 20 up-regulated genes are labelled on the graph. Among 128 differentially expressed genes, 39 genes were up-regulated and 89 genes were down-regulated in macrophages positive for CD163.

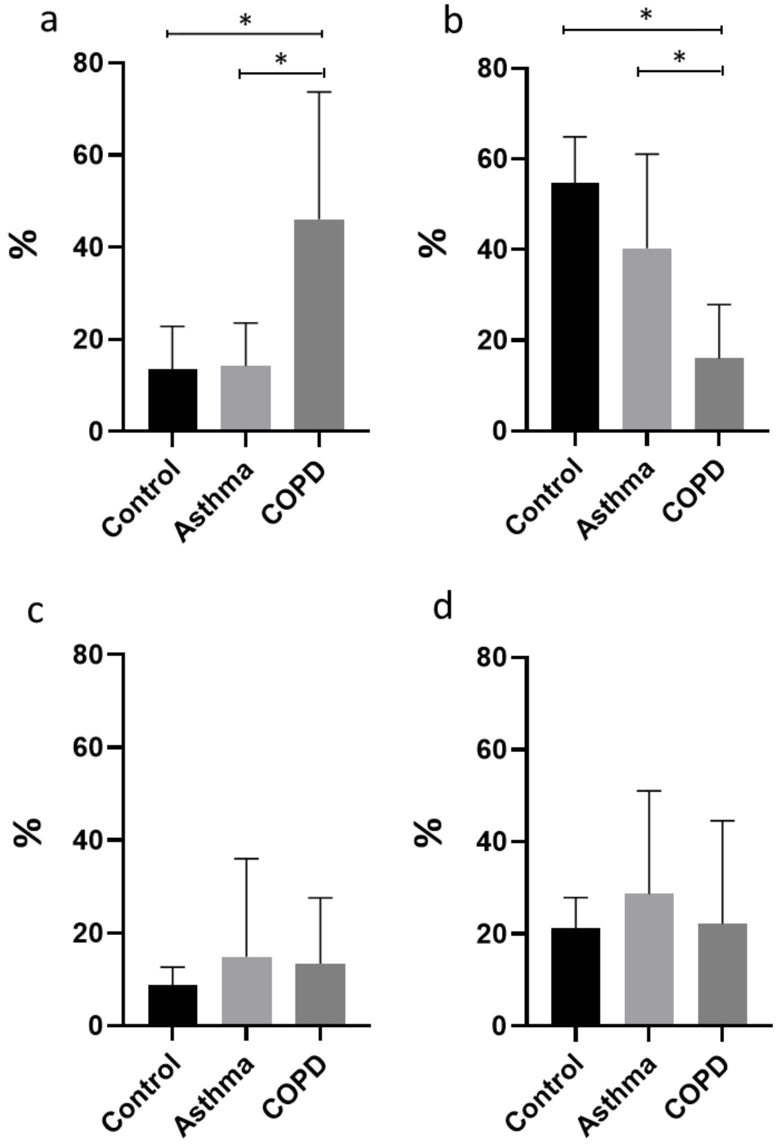

Figure S2. Macrophage sub-phenotype distributions according to disease and non-diseased states. Macrophage sub-phenotype distributions according to disease and non-diseased states. (a) Double negative (DN) subtype. (b) Double positive (DP) subtype. (c) M1 subtype. (d) M2 subtype. The proportion of DN subtype was significantly greater in COPD BAL cell pellets (46.6%, 14.7% and 13.8%, p = 0.001) compared to those of patients with asthma and those without COPD or asthma (controls). The relationship was reversed in the DP subtype (16.5% COPD; 40.8% asthma; and 55.3% controls, p = 0.002). There were no significant differences in M1 and M2 subtypes across the 3 groups. Data are presented as mean ± SD. Comparison was conducted by using One-way ANOVA test, followed by post-hoc analysis with Bonferroni test. * $p < 0.05$.

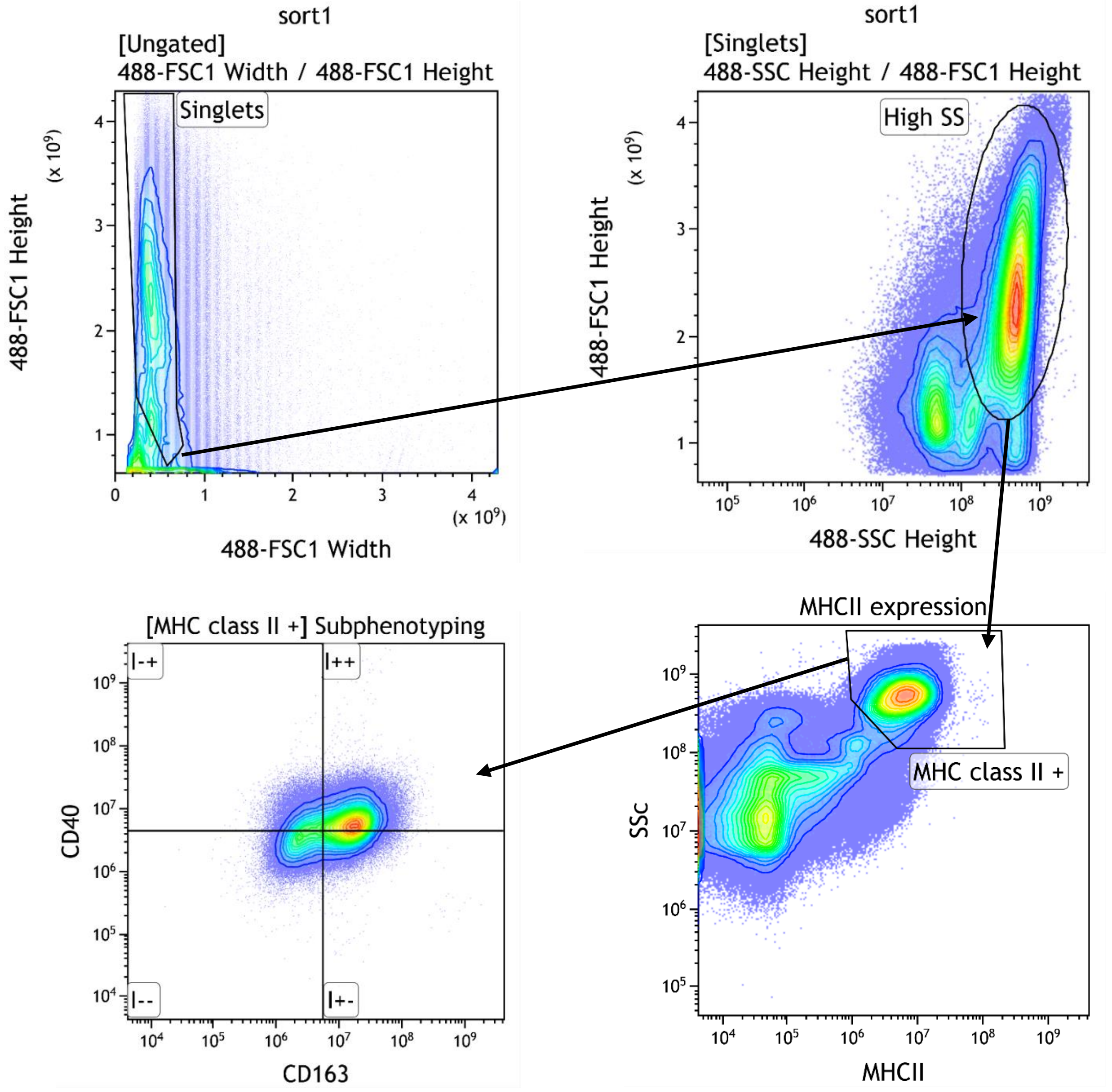

Figure S3. Representative flow cytometry panels for macrophage sub-phenotyping. Single macrophage was gated by the forward and side scatter and the presence (or absence) of human leukocyte antigen (HLA) complex in the human MHC class II. The macrophage population was double labelled with CD40 (a M1 marker) or CD163 (a M2 marker) which eventually created 4 sub-phenotypes. FSC, Forward scatter; SSC, Side scatter; High SS, High side scatter.

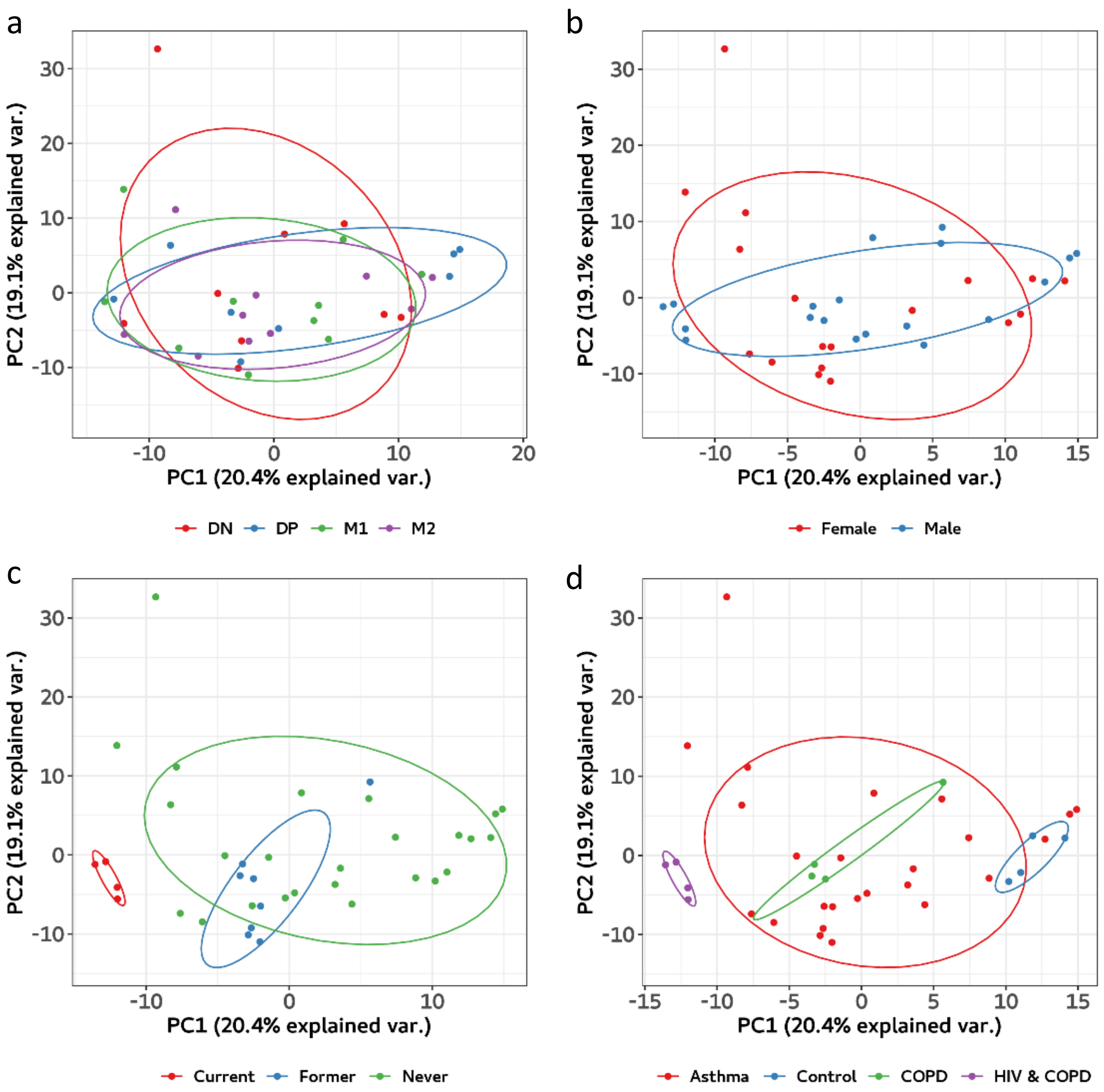

Figure S4. Principal component analysis plots colored by confounding factors: a) macrophage subtypes, b) sex, c) smoking status and d) disease status. DN, double negative; DP, double positive; COPD, chronic obstructive pulmonary disease; HIV, human immunodeficiency virus. Control are those without COPD or asthma.

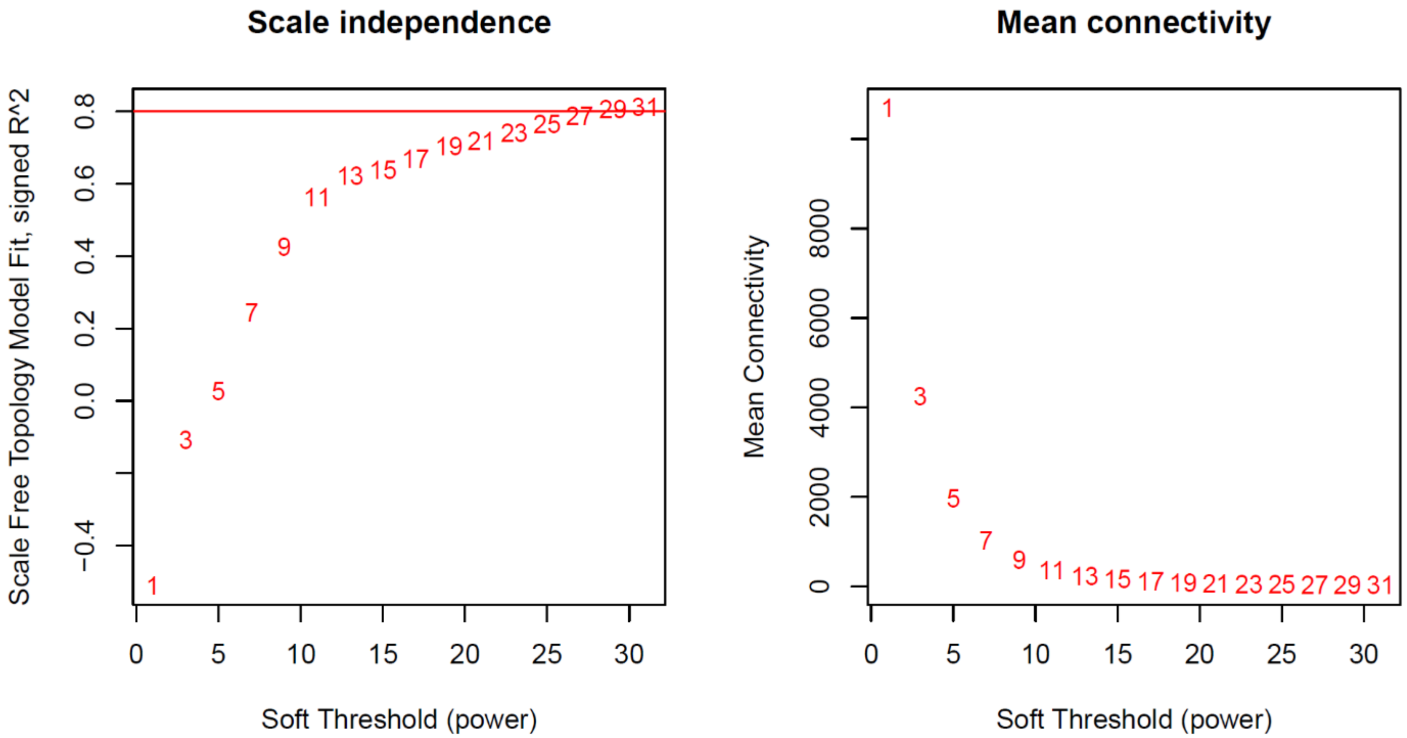

Figure S5. Weighted gene co-expression network analysis (WGCNA) diagnostic plots showing the soft-thresholding power β vs R2 and β vs mean connectivity.

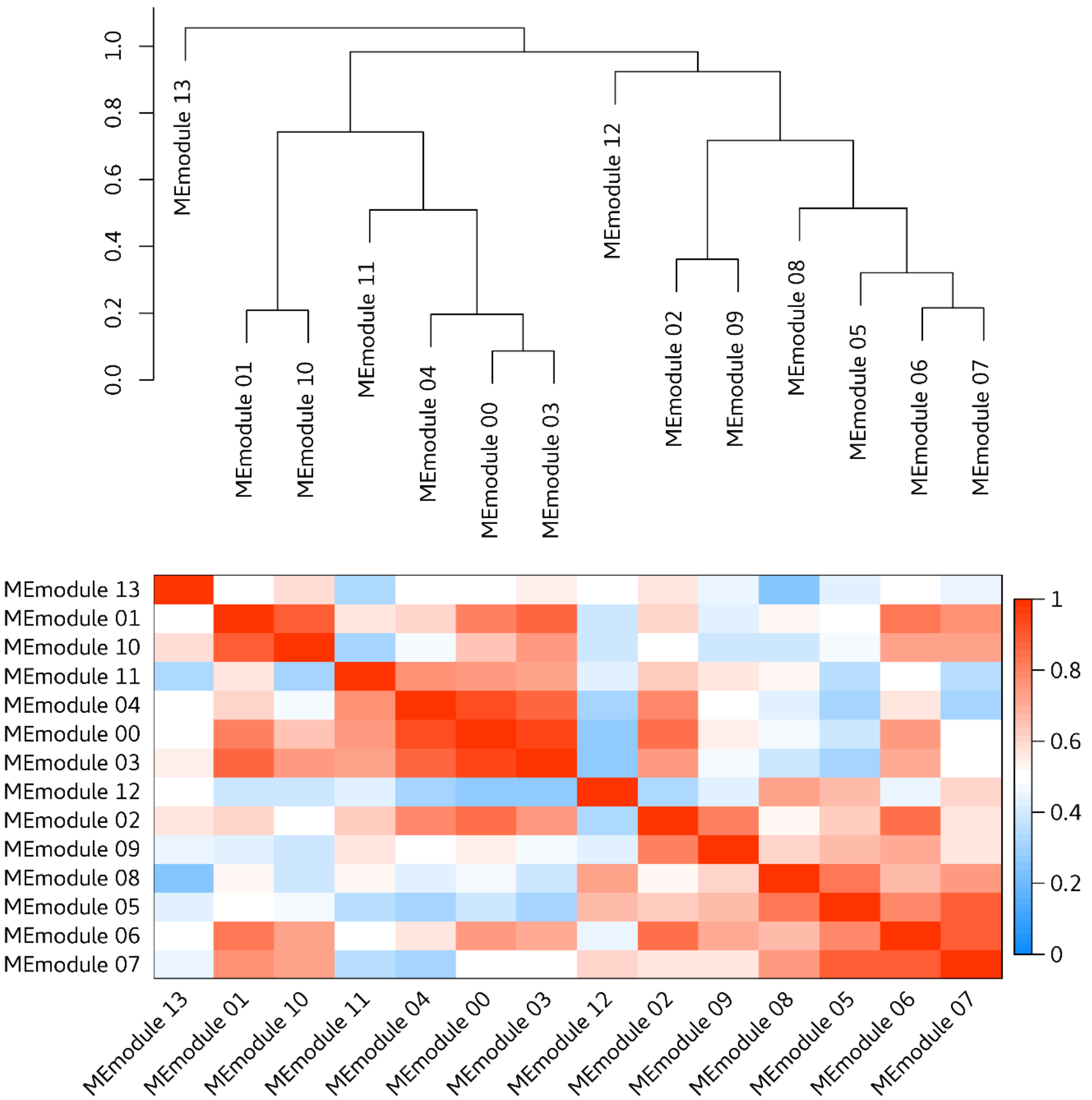

Figure S6. Module eigengene (ME) dendrogram and the corresponding heatmap showing the correlation between the MEs.
